# Trends in Motor Performance of First Graders: A Comparison of Cohorts from 2006 to 2015

**DOI:** 10.3389/fped.2017.00206

**Published:** 2017-09-29

**Authors:** Sarah Spengler, Matthias Rabel, Arvid Marius Kuritz, Filip Mess

**Affiliations:** ^1^Department of Sports and Health Science, Technical University of Munich, Munich, Germany; ^2^Department of Sports Science, University of Konstanz, Konstanz, Germany

**Keywords:** trend, children, health, motor performance, aerobic fitness, strength, speed, balance

## Abstract

**Background:**

Motor performance is an important factor for health. Already in childhood, motor performance is associated with, e.g., obesity and risk factors for cardiovascular diseases. It is widely believed that the motor performance of children has declined over recent years. However, this belief is lacking clear evidence. The objective of this study was to examine trends in motor performance of first grade students during a period of 10 years (2006–2015). We examined trends in (a) aerobic fitness, (b) strength, (c) speed, and (d) balance for boys and girls separately and considered body mass index (BMI) as a potential confounder.

**Methods:**

From 2006 to 2015, we tested 5,001 first graders [50.8% boys; mean age 6.76 (0.56) years] of 18 primary schools in Germany. Each year between 441 and 552 students of the same schools were surveyed. Performance tests were taken from the Motorik-Module Study and the “German Motor Ability Test”: “6-min run,” “push-ups,” “20-m sprint,” and “static stand.” Linear regression models were conducted for statistical analysis.

**Results:**

A slightly negative trend in aerobic fitness performance was revealed in boys (β = −0.050; *p* = 0.012) but not in girls. In the strength performance test no trend over time was detected. Performance in speed (boys: β = −0.094; girls: β = −0.143; *p* ≤ 0.001) and balance tests (boys: β = −0.142; girls: β = −0.232; *p* ≤ 0.001) increased over time for both boys and girls. These findings held true when BMI was considered.

**Conclusion:**

This study only partly supported the assumption that motor performance of children has declined: in our study, aerobic fitness declined (only in boys), while strength remained stable and speed and balance even increased in both sexes. Moreover, it seems as if BMI can explain changes in performance only to a small extent. Changed lifestyles might be a substantial cause. Further research on recent trends of motor performance and interacting variables is needed to support the results of our study and to provide more knowledge on causes of these trends.

## Introduction

Already in childhood motor performance is an important factor for health as it is associated with, e.g., obesity (1) and risk factors for cardiovascular diseases (2) in this early age. However, it is widely believed that motor performance of children has declined in recent years (3, 4). An often discussed reason for this potential decline is the verified increase of body mass index (BMI) among youth in the last decades (5, 6). The assumption that changed physical conditions are associated with changed motor performance seems to be reasonable. Though, inverse but independent trends in obesity and fitness levels among children are shown (7) and performance differences persist even after matching for overweight (8). To investigate the question of decline in motor performance among youth by reviewing the scientific literature, we first have to distinguish different aspects of motor performance. Different approaches exist (9–12). Most of them assume that motor performance is a multidimensional construct (13). The dimensionality described by Bös (12) was shown to be valid for children and adolescents (13) and assumes that motor performance can be differentiated by four main dimensions of motor performance ability which are endurance (including aerobic fitness), strength, speed, and coordination (including balance) (12).

Considering the results of extensive systematic reviews there is evidence on an improvement in aerobic fitness from 1958 until about 1970, followed by a decline from the 1970s to 2003. Strength and speed performance seemed to be stable over this time period (3, 4, 14). The aforementioned systematic reviews cover approximately five decades of the last century and, therefore, give an overview of a long period of time, in which substantial societal changes took place. Other studies examine trends over a shorter period of time, reflecting smaller societal changes, which are especially significant for the examined time period [e.g., the rapid increase of media offers within western countries in recent years (15)]. With regard to current literature about trends covering the last two decades and thus representing current societal changes, the picture is fragmentary. There are only few studies which follow heterogeneous designs and present heterogeneous results (7, 16–20): for example, the majority of studies finds a decline in aerobic fitness (7, 16, 17). One study detects a positive change in aerobic fitness, but only in girls (19). In one study, strength performance declines (20), in another study it remains stable (7), in a third study it increases (19). Trends in speed, coordination or in balance performance are less investigated. Most of the studies compare only two measurement points. The age of the examined children as well as tests performed differ between the studies considered. Most studies examine trends in boys and girls separately. This seems to be important as in some instances different results for each sex appear. However, most studies do not adjust for BMI and, therefore, cannot eliminate the possibility that changed physical conditions determine changes in performance.

The objective of this study was to examine trends in motor performance of first grade students during a period of 10 years (2006–2015). More specific, we aimed at examining trends in (a) aerobic fitness, (b) strength, (c) speed, and (d) balance, for boys and girls separately. BMI was considered as a potential confounder.

## Materials and Methods

### Study Design and Participants

Within a project of the foundation “Baden-Badener Sportstiftung Kurt Henn,” motor performance of 5,001 first grade students of 18 primary schools was tested each year from 2006 until 2015. The schools are located in southern Germany (region around Baden-Baden), while all schools in the region were represented. Each year between 441 and 552 students of the same schools took part in the survey. The survey was approved by the ethics committee of the University of Freiburg, Germany. Parent of each participant gave informed written consent before enrollment into the survey. It was conducted in accordance with the Declaration of Helsinki. The tests were performed each year in springtime. Participants’ mean age was 6.76 (±0.56) years, 50.8% were male. A detailed description of sample size, age, sex, and BMI of each cohort can be found in Table 1.

**Table 1:** Sample description.

Assessment year	*N*	Mean age in years (SD)	Male/female (%)	Mean body mass index (SD)
2006	538	6.66 (0.54)	50.9/49.1	16.53 (2.33)
2007	529	6.83 (0.53)	51.1/48.9	16.00 (2.28)
2008	552	6.70 (0.54)	51.0/49.0	15.79 (1.93)
2009	498	6.81 (0.55)	52.2/47.8	15.68 (2.15)
2010	492	6.77 (0.57)	51.0/49.0	15.70 (2.21)
2011	492	6.74 (0.58)	51.0/49.0	15.63 (2.16)
2012	494	6.81 (0.59)	46.8/53.2	16.25 (2.26)
2013	491	6.78 (0.58)	49.1/50.9	16.54 (2.63)
2014	474	6.76 (0.53)	54.4/45.6	16.12 (2.10)
2015	441	6.71 (0.54)	50.1/49.9	16.10 (2.02)

### Measurements

#### Motor Performance

Motor performance was examined using tests that are part of the Motorik-Module Study (21) and the “German Motor Ability Test” (22). Four tests were selected to cover four dimensions of motor performance: (a) aerobic fitness as part of the endurance dimension, (b) strength, (c) speed, and (d) balance as part of the global coordination dimension (12). Content-related validity of all tests was evaluated based on expert ratings with regard to significance and feasibility (rating scale ranging from 1 = “very good” to 5 = “very poor”). Values between 1.3 and 2.1 showed good content-related validity (21, 22). Reliability of all tests was good or very good (*r*_min_ = 0.73 to *r*_max_ = 0.92) (22, 23). The exact testing procedure has been described previously (13, 22, 24).

*Aerobic fitness* was tested using the test “6-min run.” Participants were asked to run or walk constantly for 6 min. The distance covered by each participant was measured by test leaders (22). *Strength* was tested using the test “push-ups”: The participant lay in prone position and the hands grasped one another on the buttocks, then placed the hands next to the shoulders and pushed his/her body up. One hand clapped onto the other, before the participant moved back to the starting position by flexing the arms. A test leader supervised correct performance and counted repetitions within 40 s (13, 22). *Speed* was tested using the test “20-m sprint.” Participants were asked to sprint a 20-m distance as fast as possible, starting in lunge position. Time was measured manually by three independent test leaders. The mean value was calculated. The better attempt out of two was used for analysis (22). The test “static stand” was used to test *balance*. The task was to stand on one leg on a T-shaped balancing bar (width 3 cm) wearing sneakers and to rest in the balance position. The number of contacts of the free foot with the ground or the T-bar (correction steps) within 1 min was counted by the test leader and was used for analysis. If the participant left the bar completely, the timer was paused until the participant was back in the initial position.

#### Anthropometrics

Height was measured with a height measuring scale (accuracy: 0.1 cm) with the participants standing upright not wearing shoes. Weight was measured by using an electronic scale (Soehnle, Murrhardt, Germany; accuracy: 0.1 kg), while participants wore sports clothes and no shoes. The measurements were performed each year by the same skilled test leaders. BMI was calculated as body mass divided by height squared (kg/m^2^).

### Data Analysis

All statistical tests were conducted using SPSS statistical software for Windows Version 23.0 (IBM Corporation, Armonk, NY, USA). Linear regression models were used to test if assessment year was a significant predictor of performance in the four motoric tests, having the year of examination as independent factor. The assumptions of linear regression were tested and confirmed. Analyses were conducted for each sex and each test separately. In a second step, multiple regression analysis was conducted to detect if the effects of assessment year were influenced by BMI, i.e., assessment year and BMI were included together as independent factors. Again, analyses were made for each sex and each test separately. For expressing temporal trends as change in percent, B coefficient of each test was multiplied by 10 to express the mean change per decade. Subsequently, we calculated the percentage of change in relation to the mean test performance. The significance level for all statistical tests was set *a priori* to α = 0.05.

## Results

**Figure 1:**
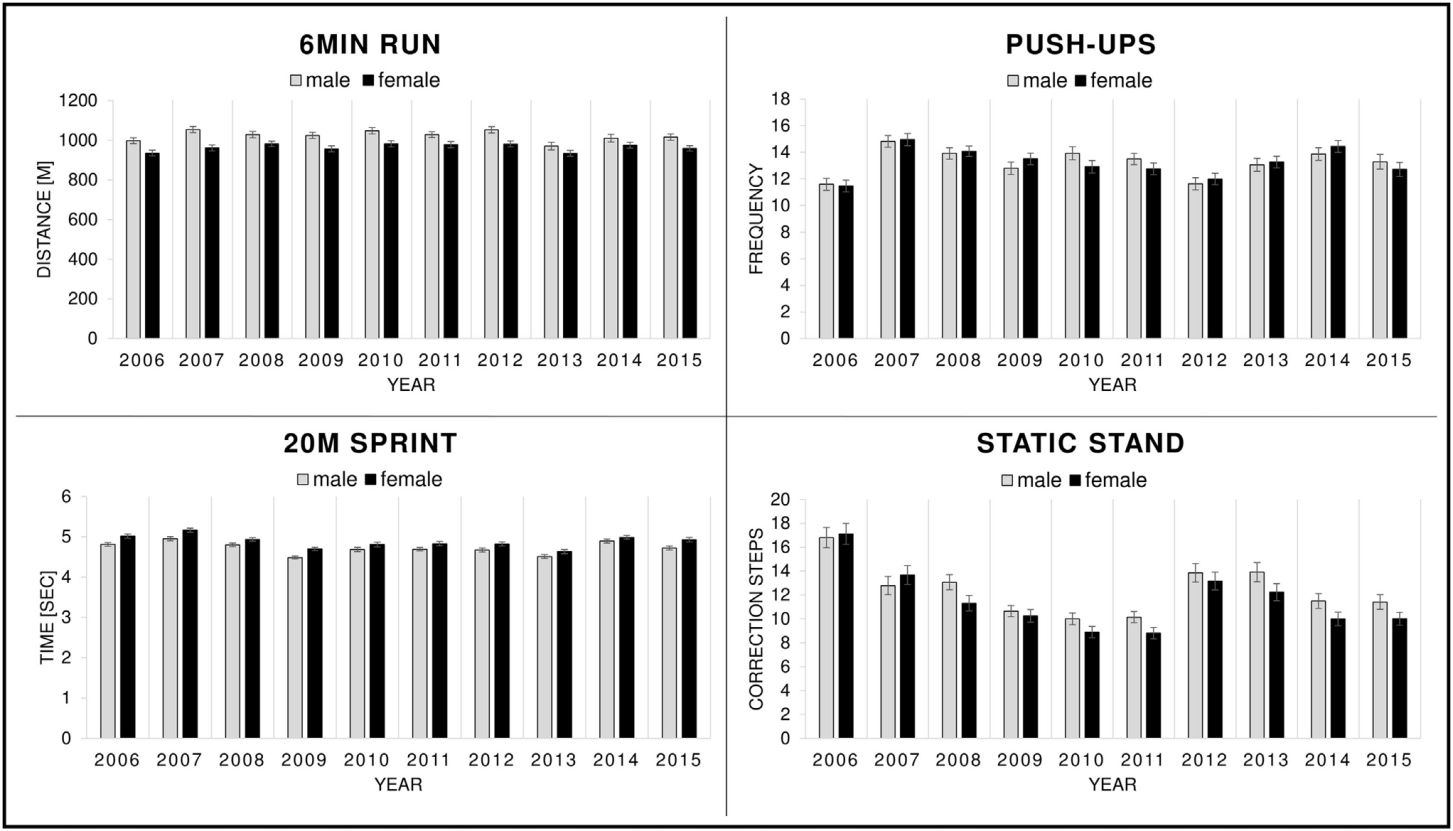
Mean values of the motor performance tests from 2006 to 2015 for boys and girls separately.

Figure 1 displays mean values of performance in (a) “6-min run,” (b) “push-ups,” (c) “20-m sprint,” and (d) “static stand” for boys and girls separately.

Table 2 shows the results of linear regression analyses, displaying the effects of assessment year on performance within the four tests.

**Table 2:** Results of linear regression analyses for boys and girls separately.

Dependent variable	Boys					Girls			
		*B* (95% CI)	β	*p*-Value	Adj. *R*^2^	*B* (95% CI)	β	*p*-Value	Adj. *R*^2^
Distance covered 6 min run (m)	Constant	5690.046 (2,033.885 to 9,346.207)				−843.739 (−4131.145 to 2443.668)			
	Year	−2.322 (−4.140 to −0.503)	−0.050	0.012	0.002	0.899 (−0.736 to 2.535)	0.022	0.281	≤0.001

Push-up repetitions	Constant	−4.882 (−111.398 to 101.633)				27.703 (−75.693 to 131.099)			
	Year	0.009 (−0.044 to 0.062)	0.007	0.739	≤0.001	−0.007 (−0.059 to 0.044)	−0.006	0.783	≤0.001

Time for 20 m sprint (s)	Constant	31.163 (20.203 to 42.123)				48.099 (36.238 to 59.959)			
	Year	−0.013 (−0.019 to −0.008)	−0.094	≤0.001	0.008	−0.021 (−0.027 to −0.016)	−0.143	≤0.001	0.020

Static stand correction steps	Constant	581.070 (425.902 to 736.238)				964.125 (805.917 to 1122.333)			
	Year	−0.283 (−0.360 to −0.206)	−0.142	≤0.001	0.020	−0.474 (−0.552 to −0.395)	−0.232	≤0.001	0.054

*B, unstandardized coefficient; β, standardized coefficient*.

In linear regression analyses, assessment year had a significantly negative effect on the distance covered within the aerobic fitness test “6 min run” in boys. Among girls, the effect of assessment year was positive but not significant. For the strength test “push-ups” no significant effect of assessment year could have been observed neither for boys nor for girls. Values within the speed test “20 m sprint” were negatively affected by assessment year in boys and even more in girls, with lower values reflecting higher performance. For the balance test “static stand” significant effects of assessment year could have been observed, too. Assessment year negatively affected the number of correction steps (reflecting a positive performance trend) in boys and in girls. Overall, the explained variance of the models was very low, with the highest value for girls in the test “static stand.”

Table 3 shows the results of the multiple regression analysis. Except for the test “push-ups,” BMI significantly predicted test performance. However, all significant effects of assessment year on test results remained nearly unchanged after including BMI into the model.

**Table 3:** Results of multiple regression analyses for boys and girls separately.

Dependent variable	Boys					Girls			
		*B* (95% CI)	β	*p*-Value	Adj. *R*^2^	*B* (95% CI)	β	*p*-Value	Adj. *R*^2^
Distance covered 6 min run (m)	Constant	5160.031 (1683.288 to 8636.775)				−503.787 (−4037.005 to 2555.367)			
	Year	−1.897 (−3.627 to −0.167)	−0.041	0.032	0.002	0.878 (−0.650 to 2.407)	0.021	0.260	≤0.001
	Body mass index (BMI)	−20.158 (−22.435 to −17.881)	−0.327	≤0.001	0.109	−18.560 (−20.460 to −16.660)	−0.362	≤0.001	0.131

Push-up repetitions	Constant	−2.521 (−109.357 to 104.314)				33.628 (−69.557 to 136.812)			
	Year	0.008 (−0.045 to 0.061)	0.006	0.759	≤0.001	−0.009 (−0.060 to 0.042)	−0.007	0.730	≤0.001
	BMI	−0.061 (−0.130 to 0.009)	−0.034	0.088	≤0.001	−0.142 (−0.206 to −0.078)	−0.088	≤0.001	0.007

Time for 20 m sprint (s)	Constant	31.619 (20.722 to 42.517)				47.186 (35.425 to 58.947)			
	Year	−0.014 (−0.018 to −0.008)	−0.097	≤0.001	0.008	−0.021 (−0.027 to −0.015)	−0.141	≤0.001	0.019
	BMI	0.023 (0.016 to 0.030)	0.126	≤0.001	0.024	0.028 (0.021 to 0.036)	0.151	≤0.001	0.042

Static stand correction steps	Constant	593.689 (440.012 to 747.366)				960.109 (804.189 to 1116.029)			
	Year	−0.292 (−0.369 to −0.216)	−0.146	≤0.001	0.020	−0.476 (−0.553 to −0.398)	−0.233	≤0.001	0.054
	BMI	0.420 (0.319 to 0.520)	0.160	≤0.001	0.045	0.467 (0.371 to 0.564)	0.184	≤0.001	0.087

*B, unstandardized coefficient; β, standardized coefficient*.

Expressing the trends of motor performance in percent, the mean change per decade in aerobic fitness performance was −2.3% (−4.0 to −0.5) in boys and 0.9% (−0.8 to 2.6) in girls. For strength performance, mean change per decade was 0.7% (−3.3 to 4.7) in boys and −0.5% (−4.5 to 3.3) in girls. For speed performance, mean change was 2.7% (1.7 to 4.0) in boys and 4.3% (3.3 to 5.5) in girls, and for balance performance, it was 22.8% (16.6 to 29.0) in boys and 41.1% (34.2 to 47.8) in girls.

## Discussion

The aim of the present study was to examine trends in motor performance of first grade students during a period of 10 years (2006–2015). Overall, we found a negative trend in aerobic fitness performance in boys but a stable performance in girls. In the strength performance test no trend over time could have been detected. Performance in speed and balance tests increased in both boys and girls. These findings held true even when BMI was considered as a confounder.

*Aerobic fitness* performance decreased in boys. This result is in line with the results of the reviews of Tomkinson and Olds (4) and Malina (14), where a negative trend of aerobic fitness between the 1970s and 2000 is shown. Moreover, the majority of the studies on trends over the last two decades also show a decline (7, 16, 17). Thus, our results support the assumption of an ongoing negative trend in aerobic fitness in boys. However, the effect (B- and β-coefficients) was small and the observed decline was smaller than in another examination from Germany, where cross-sectional data of different studies are compared (25). The divergence could be due to different methodological approaches and due to the time periods considered, as in the latter study trends between 1976 and 2005 are analyzed. In girls, aerobic fitness was stable over the 10-year period. This result is contrary to former studies which find a negative trend (4, 7, 16–18, 25). Only one study discovers a positive trend among its female sample (19). However, Tambalis et al. show in their study that performance of children living in rural areas did not change between 1997 and 2007, while performance of children in urban areas decreased (26). In our study, most of the schools where the sample was recruited are located in rural areas. With respect to the results of Tambalis et al., this might be a possible explanation for the stable performance in girls and for the relatively small decline in boys.

*Strength* performance did not change over time, neither in boys nor in girls. Stability of strength performance in both boys and girls is also shown in a large systematic review for the time period between the 1960s and 2003 (3). While evidence for trends of strength performance during this time period is quite good, trend analyses for the time period between 2000 and today are rare. We found one study by Tambalis et al. reporting a stable performance between 1997 and 2007 (7). Another study reports a positive change between 1992/1993 and 2006/2007 (19), and a third study reports a negative change between 2001 and 2006 (20). Out of these three studies, Tambalis et al. are the only ones who performed tests every year and thus offer a higher reliability. With our results we confirm their results of a stability in strength performance in children. Our results suggest that the stability holds true until today. However, this suggestion should be taken with care, as evidence is small and results showed a huge variability in strength performance for both sexes in our study.

*Speed* performance increased in both sexes over the period of 10 years. Again, our results are in line with the results of the Greek study of Tambalis et al. (7), but differ from results of the study of Dos Santos et al., where a negative change is observed for boys and girls (16). In our study, effects were relatively high compared to aerobic fitness and strength test results and the positive trend was even higher in girls. Similar findings are reported in the Greek study, where the increase in girls is also higher than in boys (7). Compared to the trend from the 1960s to 2000, where a relative stability of speed performance in children is shown (3), it seems that between 2000 and today the trend line increased and today’s children are faster than children were in earlier times.

*Balance* performance also increased in both boys and girls from 2006 to 2015. Compared to the other test results, effects in balance performance were the highest, reflecting a relatively strong positive trend in balance performance, especially in girls. Another study, comparing the balance performance of Estonian and Lithuanian adolescents finds a decline in the Estonian group, while performance in the Lithuanians increases (27). In addition, German scientists published material on changes in balance performance, comparing their results with results of former German studies. They also find an increase in the test “static stand” in their sample (28). Though, comparability of these studies is limited, as both include adolescents (11 years and older) and their methodological approach is different.

*Body mass index* predicted test performance. This result was expected, as BMI and motor performance were shown to be associated (1). However, BMI was included into the model to test if BMI was a confounder of the assessment year effects. By adding *BMI* into the regression models, effects of assessment year on test results remained significant. Actually, effects of assessment year remained almost unchanged. Tambalis et al. test the contrary hypothesis, i.e., that motor performance accounts for BMI trends in children. Likewise, after introducing the aerobic fitness variable into their model, obesity trends remain significant with practically unchanged effect sizes (7). In addition, Olds et al. report persisting performance differences after matching their sample for overweight (8). It seems as if BMI can explain changes in performance only to some extent, and our study suggested that this holds true for performance in aerobic fitness, and moreover in speed and balance. These results indicated that changed lifestyle [e.g., level of physical activity, participation in organized sports, media use, transport patterns (15)] might be a substantial cause of changes in motor performance.

Comparing the identified trends overall, our results showed that trends in the four motor performance dimensions differed. It can be speculated that these differences have at least two reasons. First, speed and balance performance could have increased due to the fact that—in contrast to most other Western countries (15)—participation rates in organized sports in young children increased within the last years in Germany (20). Moreover, participation starts at an early age with 41% of the 4-year olds and 54% of the 5-year olds being a member of a sports club (29). Organized sports programs probably focus on games and exercises suitable for young children, which are more likely to train speed and coordination instead of aerobic fitness. This could be a reason for the positive trend in speed and balance, as in former years young children were not specifically trained in such a way. Second, it is shown that everyday physical activity in children declined within recent years, as transport patterns changed from active to passive transport as well as to a more “dependent mobility” and the time playing outside decreased (15) [playing outdoors is strongly related to energy expenditure (30)]. The decrease in everyday physical activity might be a reason for the decline of aerobic fitness in boys in our study. Furthermore, in this study trends in girls were overall more positive than in boys. Similar results were found in other studies, where decreases in girls are smaller than in boys (4, 20, 25), or increases are stronger (19). Different “starting levels” could be a possible explanation: it can be assumed that motor performance in boys was on a higher relative level than in girls in earlier times. This could be due to the assumption that girls were more protected by their parents and thus played less outdoors, their games were more sedentary (e.g., playing with dolls vs. playing soccer) and boys had higher levels of overall physical activity (30). Therefore, their relative motor performance level was probably lower. So changes in lifestyle could have had less negative/stronger positive effects on their motor performance than on boys’ motor performance. In addition, it is shown that in Germany sports club participation increased more among girls than among boys (20). This could have had an impact on the more positive trends in girls, too.

The present study has some strengths and limitations that should be considered when interpreting the findings. Examinations took place in each year between 2006 and 2015, allowing the description of trends instead of single comparisons between measurement points. Moreover, data collection was region-exhaustive. However, as data are restricted to a regional sample it is not representative for Germany. Performed tests are validated and approved, but testing procedures were partly suboptimal, as the time needed for “20-m sprint” was measured manually and not with the use of a light barrier. The test “static stand” was used to measure balance. However, balance covers only one part of the complex motor performance ability dimension “coordination.” Further, comparisons with other studies should be interpreted with care, as at some stage different motor performance tests were used (e.g., “shuttle run” vs. “6-min run” to test aerobic fitness). Nevertheless, this study accounted for all four dimensions of motor performance ability (10) which contributed to a comprehensive picture of trends in motor performance of children.

In summary, this study only partly supported the widely believed assumption that the motor performance of children declines: in our study, aerobic fitness declined in boys—which is in line with other studies (4, 14)—but not in girls. All the other assessed dimensions of performance remained stable or even increased in both sexes. Strength performance remained stable. This is also shown by Tomkinson (3). In contrast, speed performance increased, while older studies present it as stable (3). Concerning balance performance, there are few studies to be found in the literature and results are heterogeneous (27, 28). In our study, balance performance increased substantially in both sexes. In addition, we showed that for all performance dimensions BMI explained changes only to a small extent. Changed lifestyles might be a substantial cause. More studies on recent trends and interacting variables are needed to support our results and to provide further knowledge on causes of these trends.

## Ethics Statement

This study was carried out in accordance with the Declaration of Helsinki. Parent of each participant gave written informed consent before enrollment into the survey. The protocol was approved by the ethics committee of the University of Freiburg, Germany.

## Author Contributions

SS conceptualized and designed the study, interpreted the data, drafted the initial manuscript, and approved the final manuscript as submitted. MR contributed substantially to conceptualization and design of the study, carried out the initial analyses, critically revised the manuscript, and approved the final manuscript as submitted. AK was involved in the data assessment, contributed substantially to analysis and interpretation of data, critically revised the manuscript, and approved the final manuscript as submitted. FM contributed substantially to conceptualization and design of the study and to interpretation of data, critically revised the manuscript and approved the final manuscript as submitted. All authors agreed to be accountable for all aspects of the work ensuring that questions related to the accuracy or integrity of any part of the work are appropriately investigated and resolved.

## Conflict of Interest Statement

The authors declare that the research was conducted in the absence of any commercial or financial relationships that could be construed as a potential conflict of interest. The reviewer TS and handling Editor declared their shared affiliation.

## References

[1] Casonatto J Fernandes RA Batista MB Cyrino ES Coelho ESMJ de Arruda M Association between health-related physical fitness and body mass index status in children. J Child Health Care (2016) 20(3):294–303. 10.1177/1367493515598645 26396021

[2] Hurtig-Wennlof A Ruiz JR Harro M Sjostrom M. Cardiorespiratory fitness relates more strongly than physical activity to cardiovascular disease risk factors in healthy children and adolescents: the European Youth Heart Study. Eur J Cardiovasc Prev Rehabil (2007) 14(4):575–81. 10.1097/HJR.0b013e32808c67e3 17667650

[3] Tomkinson GR. Global changes in anaerobic fitness test performance of children and adolescents (1958-2003). Scand J Med Sci Sports (2007) 17(5):497–507. 10.1111/j.1600-0838.2006.00569.x 17181769

[4] Tomkinson GR Olds TS. Secular changes in pediatric aerobic fitness test performance: the global picture. Med Sport Sci (2007) 50:46–66. 10.1159/0000101075 17387251

[5] Tremblay MS Willms JD. Secular trends in the body mass index of Canadian children. CMAJ (2000) 163(11):1429–33. 11192647 PMC80409

[6] Johnson W Li L Kuh D Hardy R. How has the age-related process of overweight or obesity development changed over time? Co-ordinated analyses of individual participant data from five United Kingdom birth cohorts. PLoS Med (2015) 12(5):e1001828. 10.1371/journal.pmed.1001828 25993005 PMC4437909

[7] Tambalis KD Panagiotakos DB Psarra G Sidossis LS. Inverse but independent trends in obesity and fitness levels among Greek children: a time-series analysis from 1997 to 2007. Obes Facts (2011) 4(2):165–74. 10.1159/000327994 21577024 PMC6444842

[8] Olds T Ridley K Tomkinson GR Declines in aerobic fitness: are they only due to increasing fatness? In: Tomkinson GR Olds T, editors. Pediatric Fitness Secular Trends and Geografic Variability. Basel: Karger (2007). p. 226–40. 10.1159/000101394 17387261

[9] Corbin CB A multidimensional hierarchical model of physical fitness: a basis for integration and collaboration. Quest (1991) 43:296–306. 10.1080/00336297.1991.10484032

[10] Bös K Differenzielle Aspekte der Entwicklung motorischer Fähigkeiten. [Differential aspects of the development of motor abilities]. In: Baur J Bös K Singer R, editors. Motorische Entwicklung. Ein Handbuch [Motor Development. A Manual]. Schorndorf: Hofmann (1994). p. 238–54.

[11] Fleishman EA Dimensional analysis of psychomotor abilities. J Exp Psychol (1954) 48(6):437–54. 10.1037/h0058244 13221741

[12] Bös K Handbuch Sportmotorischer Tests [Handbook of Sport-Motor Tests]. Göttingen: Hogrefe (1987). 535 p.

[13] Lämmle L Tittlbach S Oberger J Worth A Bös K A two level model of motor performance ability. J Exerc Sci Fit (2010) 8:41–9. 10.1016/S1728-869X(10)60006-8

[14] Malina RM Physical fitness of children and adolescents in the United States: status and secular change. In: Tomkinson GR Olds TS, editors. Pediatric Fitness Secular Trends and Geographic Variability. (Vol. 50), Basel: Karger (2007). p. 67–90. 10.1159/000101076 17387252

[15] Dollman J Norton K Norton L. Evidence for secular trends in children’s physical activity behaviour. Br J Sports Med (2005) 39(12):892–7; discussion 7. 10.1136/bjsm.2004.016675 16306494 PMC1725088

[16] Dos Santos FK Prista A Gomes TN Daca T Madeira A Katzmarzyk PT Secular trends in physical fitness of Mozambican school-aged children and adolescents. Am J Hum Biol (2015) 27(2):201–6. 10.1002/ajhb.22638 25284362

[17] de Moreas Ferrari GL Bracco MM Rodrigues Matsudo VK Fisberg M. Cardiorespiratory fitness and nutritional status of schoolchildren: 30-year evolution. J Pediatr (Rio J) (2013) 89(4):366–73. 10.1016/j.jped.2012.12.006 23791022

[18] Moller NC Wedderkopp N Kristensen PL Andersen LB Froberg K. Secular trends in cardiorespiratory fitness and body mass index in Danish children: the European Youth Heart Study. Scand J Med Sci Sports (2007) 17(4):331–9. 10.1111/j.1600-0838.2006.00583.x 16903897

[19] Smpokos EA Linardakis M Papadaki A Lionis C Kafatos A. Secular trends in fitness, moderate-to-vigorous physical activity, and TV-viewing among first grade school children of Crete, Greece between 1992/93 and 2006/07. J Sci Med Sport (2012) 15(2):129–35. 10.1016/j.jsams.2011.08.006 21962563

[20] Klaes L Rommel A Cosler D Entwicklung der Fitness von Kindern und Jugendlichen in Deutschland [Development of fitness of children and adolescents in Germany]. In: Klaes L Poddig F Wedekind S Zens Y Rommel A, editors. Fit sein macht Schule [“Fit sein macht Schule”]. Köln: Deutscher Ärzte-Verlag (2008). p. 29–43.

[21] Bös K Worth A Opper E Oberger J Woll A Motorik-Modul. Eine Studie zur motorischen Leistungsfähigkeit und körperlich-sportlichen Aktivität von Kindern und Jugendlichen in Deutschland. Abschlussbericht zum Forschungsprojekt [Motorik-Modul. A Study of Motor Performance and Physical Activity in Children and Adolescents. Final Report on the Research Project]. Baden-Baden: Nomos (2009). 426 p.

[22] Bös K Deutscher Motorik Test 6-18 [German Motor Ability Test 6-18]. Hamburg: Czwalina (2016). 96 p.

[23] Oberger J Romahn N Opper E Tittlbach S Wank V Woll A Untersuchungen zur motorischen Leistungsfähigkeit und körperlich-sportlichen Aktivität im Rahmen des Kinder- und Jugendgesundheitssurveys des Robert Koch-Institutes Berlin [Examinations of motor performance and physical activity within the framework of the health interview and examination survey for children and adolescents of the Robert Koch-Institute in Berlin]. In: Wydra G Winchenbach H Schwarz M Pfeifer K, editors. Assessmentverfahren in Gesundheitssport und Bewegungstherapie [Assessment Methods in Health-Related Physical Exercise and Exercise Therapy]. Hamburg: Czwalina (2004). p. 44–55.

[24] Bös K Worth A Heel J Opper E Romahn N Tittlbach S Testmanual des Motorik-Moduls im Rahmen des Kinder- und Jugendgesundheitssurveys des Robert Koch-Instituts [Test Manual of the Motorik-Module within the Framework of the Health Interview and Examination Survey for Children and Adolescents of the Robert Koch-Institute]. Wiesbaden: Bundesgemeinschaft für Haltungs- und Bewegungsförderung (2004). 41 p.

[25] Bös K Oberger J Lämmle L Opper E Romahn N Tittlbach S Motorische Leistungsfähigkeit von Kindern [Motor performance in children]. In: Schmidt WD, editor. Zweiter deutscher Kinder- und Jugendsportbericht [Second German Children and Youth Sports Report]. Schorndorf: Hofmann (2008). p. 137–58.

[26] Tambalis KD Panagiotakos DB Sidossis LS. Greek children living in rural areas are heavier but fitter compared to their urban counterparts: a comparative, time-series (1997-2008) analysis. J Rural Health (2011) 27(3):270–7. 10.1111/j.1748-0361.2010.00346.x 21729154

[27] Jurimae T Volbekiene V Jurimae J Tomkinson GR. Changes in eurofit test performance of Estonian and Lithuanian children and adolescents (1992-2002). Med Sport Sci (2007) 50:129–42. 10.1159/0000101356 17387255

[28] Klein M Emrich E Schwarz M Papathanassiou V Pitsch W Kindermann W Sportmotorische Leistungsfähigkeit von Kindern und Jugendlichen im Saarland – Ausgewählte Ergebnisse der IDEFIKS-Studie (Teil 2) [Sport-motor performance in children and adolescents from Saarland – selected results of the IDEFIKS study (part 2)]. Dtsch Z Sportmed (2004) 55(9):211–20.

[29] Woll A Jekauc D Mess F Bös K Sportengagements und sportmotorische Aktivität von Kindern [Engagement in physical exercise and sport-motor activity in children]. In: Schmidt W, editor. Zweiter Kinder- und Jugendsportbericht [Second German Children and Youth Sports Report]. Schorndorf: Hofmann (2008). p. 177–92.

[30] Sallis JF Prochaska JJ Taylor WC A review of correlates of physical activity of children and adolescents. Med Sci Sports Exerc (2000) 32(5):963–75. 10.1097/00005768-200005000-00014 10795788

